# Verticox+: vertically distributed Cox proportional hazards model with improved privacy guarantees

**DOI:** 10.1007/s40747-025-02022-4

**Published:** 2025-07-17

**Authors:** Florian van Daalen, Djura Smits, Lianne Ippel, Andre Dekker, Inigo Bermejo

**Affiliations:** 1 Department of Radiation Oncology (MAASTRO), GROW School for Oncology and Reproduction, Maastricht University Medical Centre, Maastricht, The Netherlands; 2 Department of Health Promotion, Care and Public Health Research Institute (CAPHRI), Maastricht University, Maastricht, The Netherlands; 3 Netherlands eScience Center, Amsterdam, The Netherlands; 4 Methodology, Statistics Netherlands, Heerlen, The Netherlands; 5 Data Science Institute, Hasselt University, Hasselt, Belgium

**Keywords:** Federated learning, *n*-Party scalar product protocol, Privacy preserving, Verticox, Cox proportional hazard model

## Abstract

Federated learning allows us to run machine learning algorithms on decentralized data when data sharing is not permitted due to privacy concerns. Various models have been adapted to use in a federated setting. Among these models is Verticox, a federated implementation of Cox proportional hazards models, which can be used in a vertically partitioned setting. However, Verticox assumes that the survival outcome is known locally by all parties involved in the federated setting. Realistically speaking, this is not the case in most settings and thus would require the outcome to be shared. However, sharing the survival outcome would in many cases be a breach of privacy which federated learning aims to prevent. Our extension to Verticox, dubbed Verticox+, solves this problem by incorporating a privacy preserving 2-party scalar product protocol at different stages. This allows it to be used in scenarios where the survival outcome is not known at each party. In this article, we demonstrate that our algorithm achieves equivalent performance to the original Verticox implementation. We discuss the changes to the computational complexity and communication cost caused by our additions.

## Introduction

Federated learning is a field that recently rose in prominence because of an increased focus on privacy by the general public as well as from legal bodies [1, 2]. In order to fulfill the stricter privacy requirements that were demanded by new laws such as the European General Data protection Regulation (GDPR) existing models were adapted and improved. Verticox is one such adaptation.

Verticox as described by Dai et al. [3] aims to provide a privacy preserving implementation of a Cox proportional hazards (CPH) model [4] in a vertically partitioned federated learning setting. Data is said to be vertically partitioned when the attributes are split between multiple parties. In contrast it is said to be horizontally partitioned when the records are split between multiple parties. Verticox utilizes an Alternating Direction Method of Multipliers (ADMM) framework [5] to preserve privacy. It can be used both for the training of a new Cox model as well as to classify a new individual.

However, Verticox relies on the assumption that the survival outcome is known locally at every party. This assumption is unfortunately not realistic as in vertically partitioned scenarios each attribute will normally only be locally known at one party, this includes the survival outcome.

A number of alternatives exist, such as the method proposed by Miao et al. [6] to compute CPH using cyclical coordinate descent, but it is still the case that outcome data needs to be shared with other parties. Kamphorst et al. [7] train a CPH model that uses secure multiparty computation [8] to compute log-partial likelihood at every iteration without revealing patient level data to other parties. However, the cryptographic protocols add significantly to the computational complexity and communication overhead. As such neither alternative is practical. Finally, Lu et al. [9] propose an algorithm that executes the computation of homomorphically encrypted data at a trusted third party (TTP). However, this approach introduces a single point of failure- the TTP- which could pose a significant security risk.

In this article, we propose a new extension to Verticox, which we have dubbed Verticox+. By utilizing the privacy preserving 2-party scalar product protocol [10], we avoid the assumption made in the original Verticox implementation. We will also experimentally show that the added computational complexity of using this protocol is negligible in practice.

The rest of the article is built up as follows; first, we will discuss how the original Verticox protocol works, followed by an explanation of the privacy preserving *n*-party scalar product protocol. Once both protocols have been explained we will describe the improved protocol Verticox+. We will then describe our experimental validation followed by a short discussion.

The implementation of Verticox+ is available on GitHub^1^ and has been designed to work with the vantage6 federated learning framework [11].

## Background

In the following subsections we will discuss the background of our solution. First, we will introduce Verticox, and then we will introduce the scalar product protocol.

### Verticox

Verticox is a decentralized version of the Cox proportional hazards regression model where covariates can be distributed over multiple data sources. The parameters are computed without sharing raw data between the parties and the resulting model is equivalent to a centralized version of a Cox model. The original algorithm achieves this by decomposing the original optimization problem for Cox proportional hazards into subproblems that can be solved separately. This provides a layer of protection for the data against honest-but-curious adversaries with access to any of the clients or central server.

The Verticox algorithm first estimates the parameters at the client-side based on the covariates that are available locally to each party. Next, an aggregation of these results is sent to a central server, which combines the results of the various parties, and further optimizes the parameters. The updated values are then passed back to the parties at the start of a new iteration. For a more detailed description of the exact techniques used we refer to reader back to the original paper [3].

### Scalar product protocol

In order to solve the privacy issues that are present in the original Verticox algorithm, we use can use a privacy preserving scalar product protocol. The privacy preserving scalar product protocol is an important building block in many federated data analysis and machine learning applications. There exist several variants [10, 12–17]. Our proposed protocol requires the use of a 2-party privacy preserving scalar product protocol when dealing with vertically split data. Furthermore, we noticed that a future potential adaptation to a hybrid, that is to say both horizontally and vertically, split scenario would require a *n*-party protocol. To be ready for this potential future adaptation, we choose to utilize the *n*-party protocol variant [12] in our implementation, which is based on the 2-party protocol proposed by Du and Zhan [10]. This method introduces a third party, labeled the commodity server, to generate the random vectors that are used to encrypt the data. This server does not participate any further in the computation.

As we will be focused on vertically split scenarios we will only describe the 2-party scenario in this paper. The 2-party algorithm by proposed by Du and Zhan works as follows:

#### The protocol

There are two parties, Alice and Bob. Alice has a vector $A$ and Bob has another vector $B$, both of the vectors have $n$ elements. Alice and Bob want to compute the scalar product between $A$ and $B$, such that Alice gets $V_1$ and Bob gets $V_2$, where $V_1 +V_2 = A \cdot B$ and $V_2$ is randomly generated by Bob. Namely, the scalar product of A and B is divided into two secret pieces, with one piece going to Alice and the other going to Bob. We assume that the following computation is based on the real domain.

1. A Trusted Third Party (TTP) server generates two random vectors $$R_a$$ and $$R_b$$ of size $$n$$, and lets $$r_a + r_b = Ra \cdot Rb$$, where $$r_a$$ (or $$r_b$$) is a randomly generated number. Then the TTP sends $$(R_a,r_a)$$ to Alice, and $$(R_b,r_b) $$to Bob.
2. Alice sends $\hat{A} = A + R_a$ to Bob, and Bob sends $\hat{B} = B + R_b$ to Alice.
3. Bob generates a random number $V_2$, and computes $\hat{A} \cdot B +( r_b - V_2)$, then sends the result to Alice.
4. Alice computes $$( \hat{A} \cdot B +( r_b - V_2)) - (R_a \cdot \hat{B} )+ r_a = A \cdot B - V_2 +(r_b - R_a \cdot R_b + r_a) = A \cdot B - V_2 = V_1 $$

For a more detailed description of the exact techniques used we refer to reader back to the original paper [12].

## Verticox+

Verticox+ is an extension of Verticox that no longer a requires sharing the survival outcome data with all parties involved. By making use of the scalar-product-protocol, we have been able to isolate the outcome data to the aggregation server. We make a slight modification to the original algorithm to incorporate the scalar product protocol. Table 1 explains the notations that will be used throughout the reminder of the article.

**Table 1:** Notation

Notation	Description
*K*	Total number of parties
*N*	Total number of records
$\beta _{k}$	Coefficients at party *k*
*T*	Number of distinct event times
$$t_{n} $$	Distinct event time of patient *n*
*p*	Index of iteration
$\rho$	Penalty parameter of ADMM method
*z*	Auxiliary variable
$E_{t}$	The index set of records with observed events
$x_{nk}$	The local feature value at index *n* for party *n*

The original Verticox pseudocode can be summarized as found in Algorithm 1. The full pseudocode can be found in original paper by Dai et al.. Note the emphasis on the requirement that outcome data $[E_1, ... E_T]$ is present at every party.
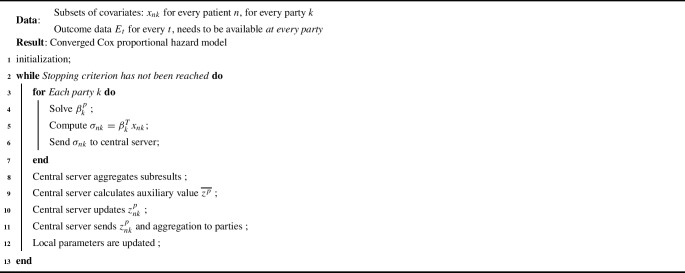


The main privacy issue lies within solving $\beta _{k}^{p}$. This is done using Eq. 1

1 $$\begin{aligned} \begin{aligned} \beta _{k}^{p}&=\begin{bmatrix} \rho \displaystyle \sum _{n=1}^{N} x_{nk}^{T}x_{nk} \end{bmatrix}^{-1} \cdot \\&\qquad \begin{bmatrix} \displaystyle \sum _{n=1}^{N} (\rho z_{nk}^{p-1} - \gamma _{nk}^{p-1} ) x_{nk}^{T} + \sum _{t=1}^{T}\sum _{n\in E_{t}}x_{nk} \end{bmatrix} \end{aligned}\nonumber \\ \end{aligned}$$

The problem lies in the last part of the equation: $\sum _{t=1}^{T}\sum _{n\epsilon E_{t}}x_{nk}$. This part has a reference to $E_{t}$, which is the index set of samples with an observed event at time *t* . Therefore, for every time *t* we need to select the samples with an observed event. This requires the availability of outcome data at every party. In real-world use cases, this is not always possible.

Verticox+ will solve this problem by making use of the scalar-product-protocol. To do that, we translate the inner sum $\sum _{n\epsilon E_{t}}x_{nk}$ to a scalar product: $u_{kt} =x_{k} \cdot \overrightarrow{(E_{t})}$

In this case, $\overrightarrow{(E_{t})}$ is the Boolean vector of length *N* that indicates for each sample whether it had an event at time *t* (indicated as 1) or not (indicated as 0). $\beta _k^{p}$ will now be solved according to Eq. 2.

2 $$\begin{aligned} \begin{aligned} \beta _{k}^{p}&=\begin{bmatrix} \rho \displaystyle \sum _{n=1}^{N} x_{tnk}^{T}x_{tnk} \end{bmatrix}^{-1} \\  &= \begin{bmatrix} \displaystyle \sum _{n=1}^{N} \left( \rho z_{nk}^{p-1} - \gamma _{nk}^{p-1} \right) x_{nk}^{T} + \sum _{t=1}^{T} u_{kt}\end{bmatrix} \end{aligned}\nonumber \\ \end{aligned}$$

Since $u_{kt}$ per time *t* stays constant over iterations, we will only need to compute this once at the initialization step. The rest of the algorithm will remain the same. Additionally, as this can be resolved independently for each feature, and it is known that the data is vertically split we know that even if more than 2 parties are involved in the complete analysis, only 2 parties are involved for computing $u_{kt}$ at a single institution *k*, which limits the computational complexity introduced by its use.

**Fig. 1:**
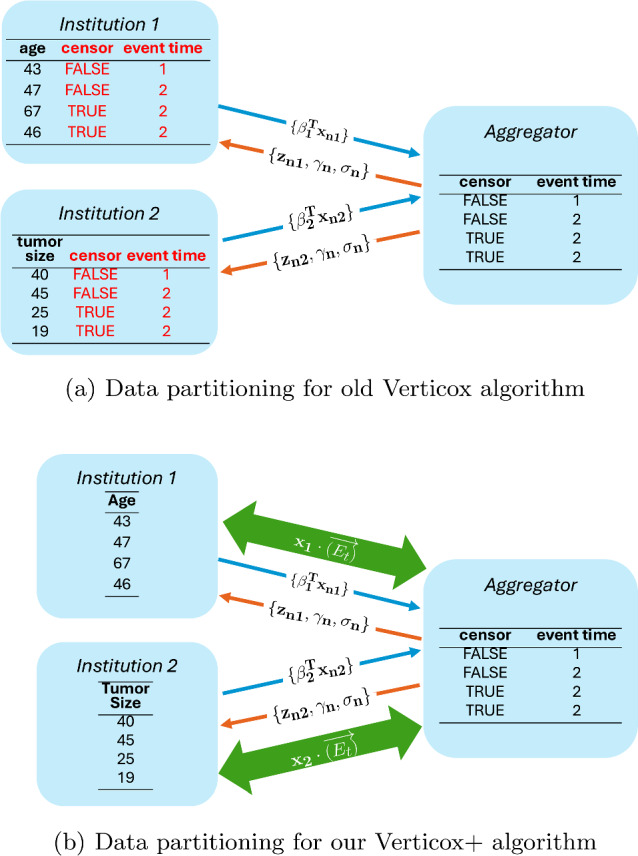
In the original Verticox algorithm, the survival outcome (event time, censored) is required to be available at all parties (**a**), while in our Verticox+ algorithm, the survival outcome only needs to be at the central aggregator server (**b**)

**Fig. 2:**
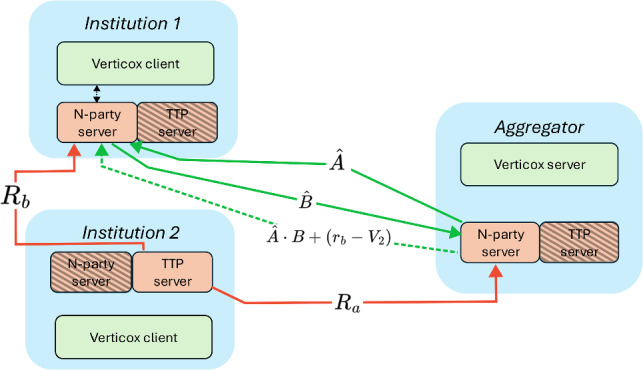
When an institution needs to compute $U_{kt}$ it will perform the 2-party scalar product protocol together with the aggregator server. The other institution will take the role of TTP

A summary of the updated Verticox+ algorithm can found in Algorithm 2:
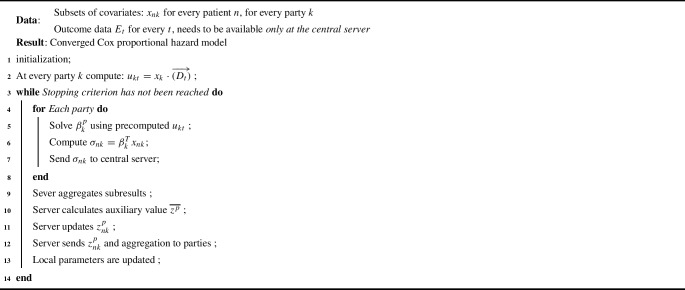


In the updated version of the algorithm, there is no longer a need to share outcome data with all collaborating parties. Figure 1a show that while the algorithm only sends aggregations back and forth between parties, it requires the outcome data (censor and event time) to be present at every institution. Figure 1b show that this is not necessary any more. When a party needs to compute $u_{kt}$ using the 2-party scalar product protocol it exchanges the values as shown in Fig. 2. We are making use of the implementation of the n-party scalar product protocol, which is equivalent to the 2-party protocol when combining data from 2 parties. The figure also shows that when one of the institutions needs to combine data with the aggregator (where the outcome data is located), the other institution can be used as a trusted third party. Institutions are switching roles between n-party server and TTP server depending on which party needs to calculate $u_kt$.

## Time complexity & communication overhead

In this section we will discuss the time complexity and communication overhead of Verticox+. We will start by discussing these aspects of Verticox, to provide a baseline. Afterwards we will discuss the time complexity of the *n*-party scalar product protocol. Finally, we will discuss the consequences of combining these two protocols.

### Time complexity

Let us consider how the addition of the scalar product protocol affects the time complexity. Since the scalar product protocol has only been used at the client side, the time complexity at the server side will remain $O(N^{3} )$, which is the complexity of the Newton–Rhapson optimization.

At the client side, the original computational complexity was determined by generating and inverting matrix $\sum _{n=1}^{N}x_{nk} x_{nk}^{T}$ are $O(NM_{k}^{2})$ and $O(M_{k}^{3})$ respectively. Our adaptations add in the scalar product protocol which is $O(N^{2})$. In practice though we will see that the main bottleneck can be found in the aggregation server.

### Communication cost

The original Verticox sends intermediate values $z_{nk}$, $\overline{\gamma }_{n}$, and $\overline{\sigma }_{n}$ from the central server to the clients at every iteration. In turn, the clients send back $\overline{\sigma }_{nk}$. This results in a communication cost of 4*NK*. Additionally, the scalar product protocol has a cost of 4*N*, which will need to be run for every institution, which leads to a cost of 4*NK*. However, the scalar product protocol is only run once per analysis, while the intermediate values are communicated every iteration. This leads to a communication cost of $4NK + I * 4NK$, where *I* stands for the number of iterations for convergence.

### Fixed precision

The *n*-party scalar product protocol is designed to work using integer values. However, within Verticox+ it will be used to calculate results that depend on floating point values. In order to make these values useable within the scalar product protocol we will make use of fixed-point precision. The values will be scaled by a fixed factor; this factor corresponds to the required precision (e.g. the value will be scaled by a factor 10, 000 when working with a fixed precision of 5 decimals). Once the scalar product protocol has finished the final result will be scaled back to the desired precision.

This fixed precision approach makes it viable to use the scalar product protocol even when it is necessary to work with floating point values. In principle any level of precision can be chosen, however there will be a trade-off; a greater precision will result in larger numbers being used in the scalar product protocol. This can create technical problems when it results in a number overflow error. Additionally, numbers with more digits will take longer to multiply. As such, a high precision will eventually affect the runtime performance of Verticox+. However, we experimentally determined that a fixed precision of 5 decimals is sufficient for most purposes. Furthermore, we expect the effect on the total runtime of Verticox+ to be minimal as the bottleneck is outside of the part that utilizes the scalar product protocol.

In addition to this we would like to note that fixed precision could potentially be used to further improve privacy guarantees. A lower precision can obfuscate records containing values that are unique at a higher level of precision. Its exact potential warrants further investigation in future research.

## Experimental validation

We ran several experiments to validate our method. We implemented the algorithm in Python and Java, and ran the parties in separate Docker containers. We used a single virtual machine with Ubuntu 22.04, 8 cores with a clock speed of 1996.250 MHz and 32 GB RAM running in SURF research cloud, which is part of the Dutch national research infrastructure. As data we used part of the SEER dataset [18]. The parameters of the algorithm that we kept fixed can be found in Table 2.

**Table 2:** Experimental parameters

Parameter	Fixed value
Penalty parameter $\rho$	0.25
Fixed precision of *n*-party protocol	5
Newton–Raphson precision	0.00001

**Fig. 3:**
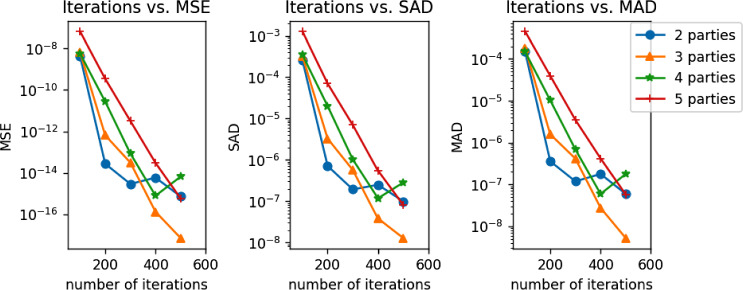
The MSE, SAD, & MAD scores of Verticox+

We ran 3 different experiments. In the first experiment, we fixed the number of records to 100 and varied the number of parties and iterations to see how that will affect runtime and accuracy. Accuracy has been measured in 4 different ways. In theory, adding the *n*-party protocol to the original Verticox algorithm will introduce inaccuracy into the model because the values need to be expressed in fixed-point precision. To test whether this is true in practice we ran our implementation of the original Verticox algorithm with the same parameters. We use c-index [19] to compare the predictions of the model to the ground truth. Additionally, we used 3 metrics to compare the resulting coefficients against ones that have been computed by a central Cox proportional hazards model. For this, we compute mean squared error (MSE), summation of the absolute difference (SAD), and maximum absolute difference (MAD). As can be seen in Table 3 the accuracy of the central model is identical to the accuracy of Verticox+. This is because the variables in the SEER dataset require limited precision, since they consist of values with no more than 2 digits. Looking at MSE, SAD and MAD (Fig. 3), we can see that the difference between Verticox+ and a Cox proportional hazards model learned on centralized data diminishes after a few hundred iterations.

**Table 3:** Performance of central Cox proportional Hazards vs. Verticox+ algorithm

Parties	Iterations	Preparation runtime	Convergence runtime	mse	sad	mad	c-index verticox+	c-index central Cox model
2	100	5.349767	80.217799	4.2080e–09	2.5862e–04	1.4883e–04	0.634463	0.633898
	200	5.254844	128.451124	2.7379e–14	7.1284e–07	3.5788e–07	0.633898	0.633898
	300	5.389899	162.827923	2.9245e–15	1.9241e–07	1.1896e–07	0.633898	0.633898
	400	5.309092	200.631222	5.8759e–15	2.4782e–07	1.7857e–07	0.633898	0.633898
	500	5.298918	225.582957	7.2622e–16	9.8687e-008	5.9357e-008	0.633898	0.633898
	1000	5.308558	394.353162	6.4518e–16	7.4555e-008	6.1778e-008	0.633898	0.633898
3	100	6.050240	82.893001	6.5099e-009	3.2313e-004	1.8406e-004	0.633616	0.633898
	200	6.113631	142.582830	6.4638e–13	3.2763e-006	1.5817e-006	0.633898	0.633898
	300	6.125730	180.786434	3.1175e–14	5.6994e-007	4.1506e-007	0.633898	0.633898
	400	6.172221	214.349878	1.3309e–16	3.7855e-008	2.7629e-008	0.633898	0.633898
	500	6.029485	245.121609	7.1598e–18	1.2724e-008	5.2342e-009	0.633898	0.633898
	1000	6.105557	408.771761	1.3322e–16	3.7714e-008	2.7629e-008	0.633898	0.633898
4	100	6.886146	84.721814	5.6519e-009	3.4969e-004	1.4831e-004	0.633898	0.633898
	200	6.924360	141.273931	2.7149e–11	2.0121e-005	1.0284e-005	0.633898	0.633898
	300	6.832369	197.663962	8.5444e–14	1.0257e-006	6.8763e-007	0.633898	0.633898
	400	6.901601	228.786964	7.9107e–16	1.1789e-007	5.9357e-008	0.633898	0.633898
	500	6.751254	257.553203	6.5969e–15	2.8261e-007	1.7857e-007	0.633898	0.633898
	1000	6.864445	417.910666	6.0346e–16	7.5356e-008	5.9357e-008	0.633898	0.633898
5	100	7.704099	85.908523	6.4246e-008	1.2748e-003	4.4972e-004	0.634181	0.633898
	200	7.732730	139.656296	3.4136e–10	7.0454e-005	3.9577e-005	0.633898	0.633898
	300	7.673841	191.090284	3.1704e–12	6.9955e-006	3.4890e-006	0.633898	0.633898
	400	7.703514	239.151800	3.0157e–14	5.4475e-007	4.1941e-007	0.633898	0.633898
	500	7.698904	266.683773	5.7475e–16	8.0196e-008	5.7432e-008	0.633898	0.633898
	1000	7.672942	414.228958	6.0121e–16	7.0263e-008	5.9852e-008	0.633898	0.633898

The second experiment evaluates how runtime scales with increasing number of covariates (features) in the model. Again, we fixed the number of records to 100 and the number of iterations to 500. The number of parties has been fixed to 3. We evaluated the algorithm runtime from 2 and up to 10 features.

**Fig. 4:**
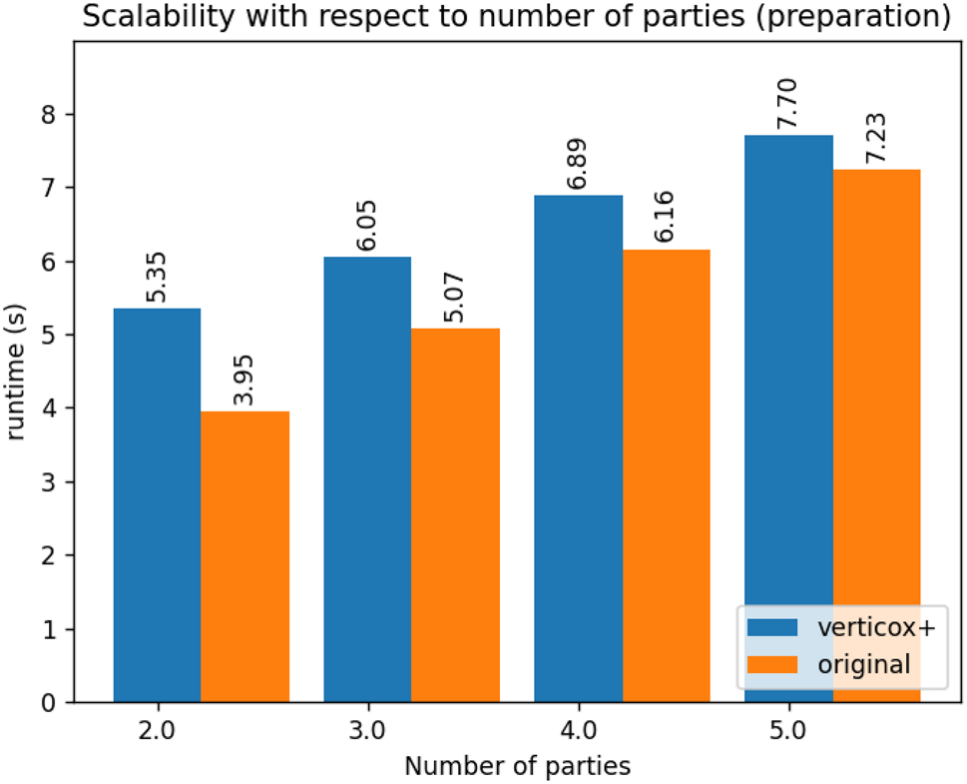
Comparison between Verticox+ and Verticox of the runtime duration of the preparation phase

**Fig. 5:**
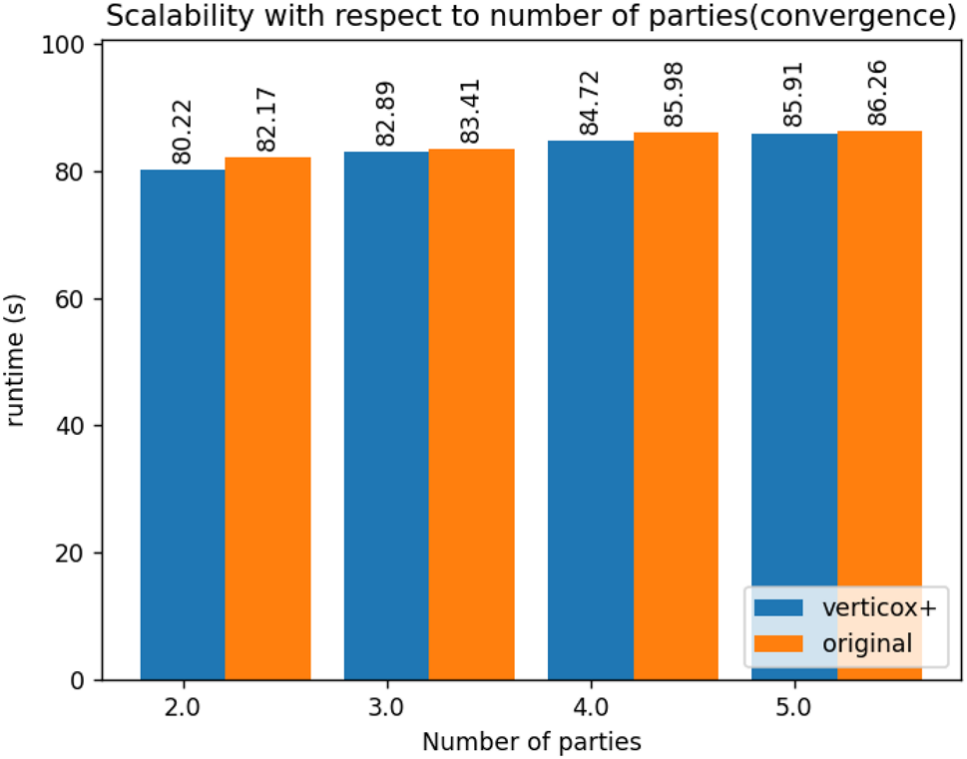
Runtime duration of Verticox+ with various numbers of parties

**Fig. 6:**
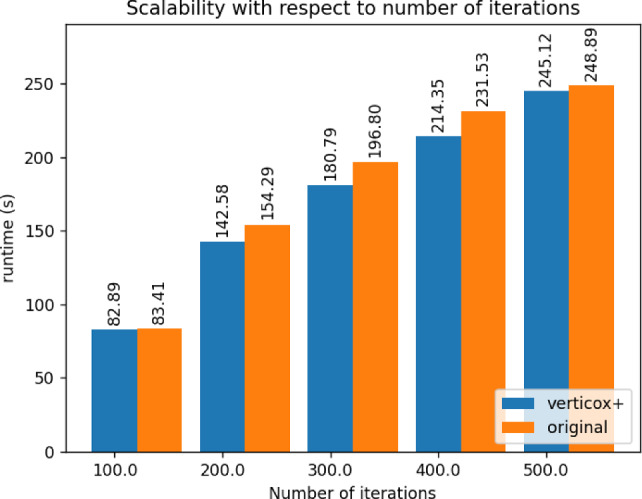
Runtime duration of Verticox+ with various numbers of maximum iterations

**Fig. 7:**
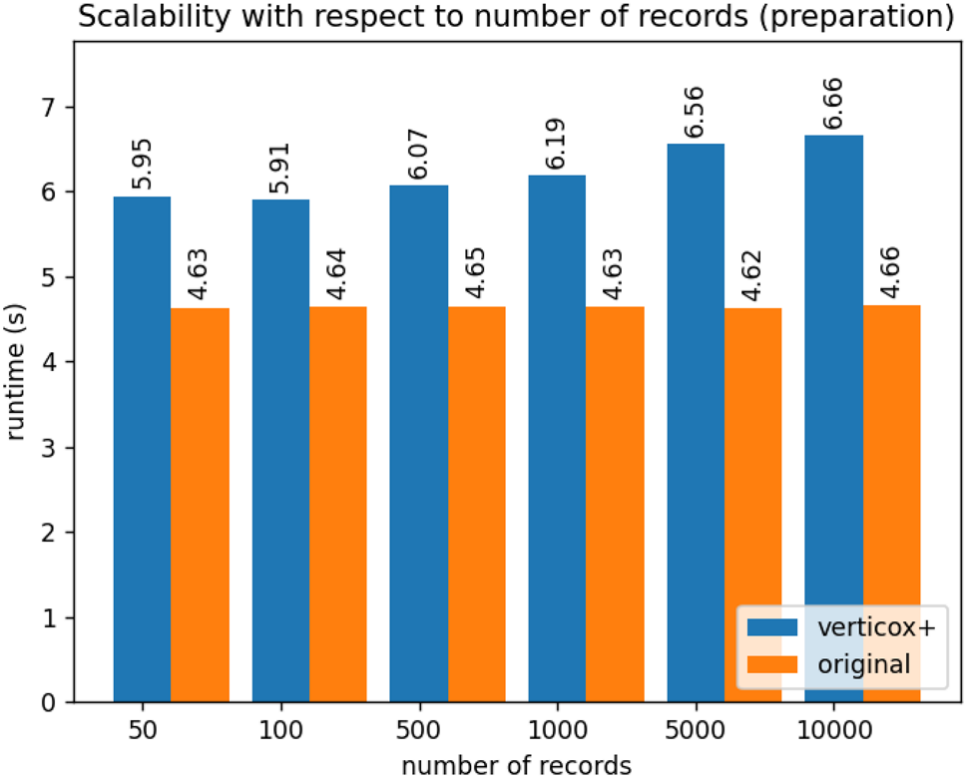
Runtime duration of the preparation phase of Verticox+ using various numbers of records

**Fig. 8:**
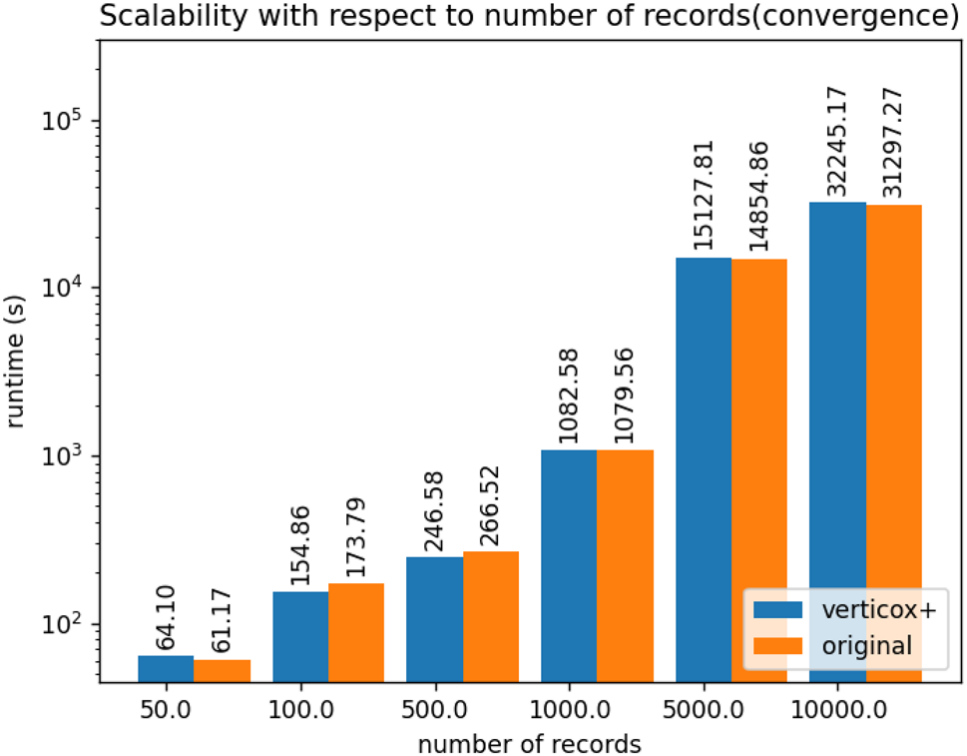
Runtime duration of the convergence phase of Verticox+ using various numbers of records

**Fig. 9:**
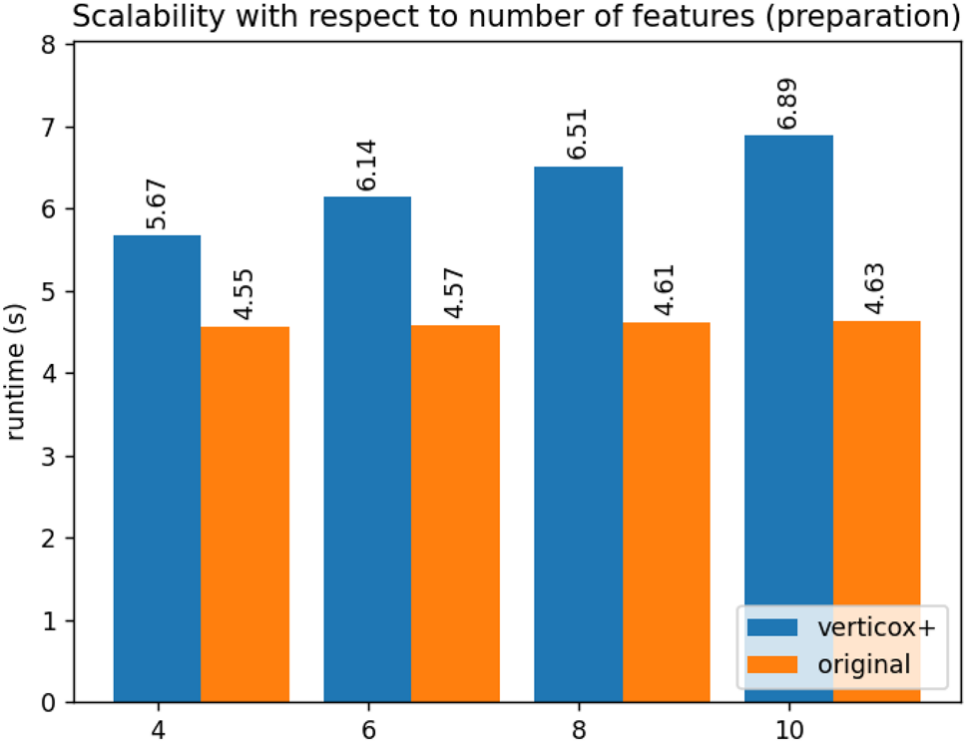
Runtime duration of the preparation phase of Verticox+ using various numbers of features

**Fig. 10:**
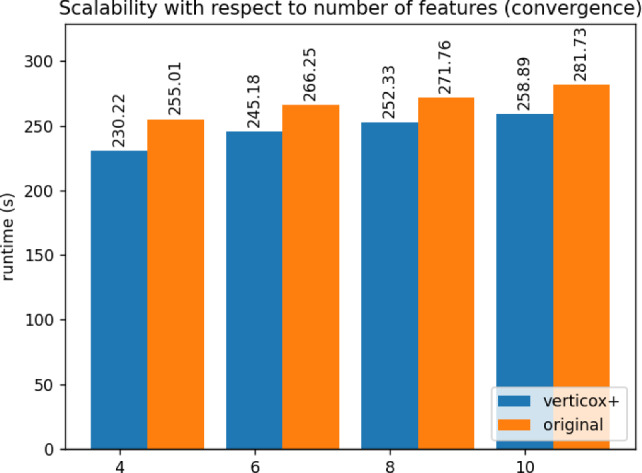
Runtime duration of the convergence phase of Verticox+ using various numbers of features

As can be seen in Figs. 4, 5, and 6, our addition of the scalar product protocol does not negatively affect the runtime. In fact, Verticox+ even has a shorter runtime for preparation as the number of parties increases. This is unexpected and likely relates to the implementation details of the *n*-party protocol. While we reimplemented the original Verticox algorithm in Python, the *n*-party protocol was actually implemented in Java. Since Java is a compiled language, it generally performs faster than the interpreter language Python. In the end, the bottleneck will not be in the preparation time, but rather in runtime of the main part of the algorithm where the model converges. In this part, Verticox+ performs the same as its predecessor (see Figs. 7 and 8).

In the third experiment we fixed the number of iterations to 500 and the number of parties to 3. We set the number or records to 50, 100 or 500 and timed the runtime. As can be seen in Figs. 9 and 10, the number of records does not affect the runtime significantly during the preparation phase. Convergence runtime is affected by the number of records, but not more than the original verticox.

## Discussion

With the addition of the scalar product protocol to the Verticox algorithm, all data used for the analysis can stay at the source, including outcome data. Moreover, the addition of this protocol, which is potentially heavy in terms of computation, did not add significantly to the total runtime in our experiments. Neither does the additional overhead introduced by the fixed-point precision. This is because the bottleneck of the computation lies within the Newton–Raphson optimization from the original algorithm. This indicates that Verticox+ is a viable extension of the original algorithm.

There are still a couple of security issues to consider though. The Verticox+ algorithm shares record-level aggregations with the central server. That is, in every iteration the parties share their risk estimates for every record with the central server. Although this is not raw data, it is still patient level information. Additionally, it can be viewed as relatively sensitive data as it represents the risk of a given disease for a specific patient. Direct access to this information could be problematic if it falls in the wrong hands.

However, by placing the server in the care of the party that already owns the survival outcome data the practical risk is limited. Providing this party with the risk scores minimizes the privacy concerns as this party already knows the survival outcome this risk score represents, and thus would not learn anything new. This limits the risk of direct access to the risk score.

Access to the risk estimates in each iteration also opens an additional possible attack [20–22]. The aggregating party could attempt to reverse engineer the training data belonging to each other party based on the intermediate values revealed between the iterations. However, this does require the aggregating party to know which attributes are present at each other party. Additionally, reversing this information becomes more complex as the number of attributes at the other party grows.

This privacy concern could be mitigated by moving the central aggregation away from the outcome datasource and performing the central aggregation on a “neutral” server provided by a trusted third party. The outcome data would have to be queried by the central server using the scalar product protocol. Unfortunately, this means that the scalar product protocol would have to be run in every iteration, instead of only during the preparation phase. The concern is that this will add a significant increase to the total runtime. Adding this protocol with complexity $O(N^{2})$to the Newton–Rhapson optimization $(O(N^{3}))$ will turn it into a complexity of $O(N^{4})$. Additionally, the *n*-party protocol will add a constant communication overhead to this part of the computation (*O*(6)). Although this overhead is constant, in practice the communication overhead is the bigger bottleneck when compared to the computational cost, and will add significantly to the total duration of the algorithm.

A more practical solution may be to mandate the use of a framework like Vantage6, which provides an infrastructure that explicitly limits what the aggregating party is able to do by only allowing pre-approved Docker images with vetted code to be executed. By explicitly creating this limitation, the various parties involved can establish a sufficient level of trust that no data will be leaked.

This risk, and the limitations imposed by the time complexity of the technical solutions, highlight the need for a comprehensive legal and infrastructure solutions to augment the technical privacy preserving solutions in any real world project. This also means that Verticox+ is best used in a setting where such things can viably be implemented. Implementing such solutions, and establishing the required level of trust, is difficult in an open internet of things setting, where any party is free to join. However, in a formal research setting this is indeed viable.

The scalar product protocol brings one additional privacy concern compared to the broader Verticox+ protocol. It requires a trusted third party, which can generate secret shares and aggregate the intermediate results of the protocol. However, in comparison to Lu et al. [9], the TTP has reduced privileges and does not need to process any data directly. This reduces the risks of using a TTP considerably. Additionally, similar to the previous concerns, framework such as Vantage6 is an excellent solution to set up the necessary infrastructure to ensure the reliability of the trusted third party, further limiting the potential risks.

## Future work

There are currently three major limitations that we would like to improve upon. The current implementation of Verticox+ has not been made to deal with a hybrid split in the data, that is to say a split that is partially horizontal and partially vertical. While certain parts, such as the scalar product protocol, do not need any additional work to fit in a hybrid setting, we need to determine if it is possible to use the algorithm as a whole in a hybrid setting.

Secondly, the role of aggregator currently befalls to the party that owns the outcome data. If the role of aggregator could be moved to a neutral party without data, it would not know which records the intermediate values are linked to. This lowers the risk of data leaking. Lastly, we wish to improve the runtime complexity of the optimization step, for example by using a different faster optimization algorithm. This step is currently a considerable bottleneck in the algorithm, and improving it would lead to significant gains in terms of the running time of the algorithm.

## Conclusion

In this paper, we have provided an extension to the original Verticox protocol that we dub Verticox+. The original protocol allows the user to train a Cox Proportional Hazard model in a vertically partitioned federated setting. However, the original algorithm relies on the assumption that every party involved has access to the survival outcome for each record. This is unrealistic in a vertical scenario and would most likely require this survival outcome to be shared, which represents a serious privacy concern as the survival outcome used to train a Cox proportional hazard model represents a sensitive attribute, such as a hospitalization event or death due to a certain disease. Verticox+ removes the need for this assumption by using the scalar product protocol to perform the relevant calculations in a privacy preserving manner.

Our experiments show that Verticox+ achieves comparable performance to both Verticox and a centrally trained model. This indicates Verticox+ works as intended. Additionally, our experiments show that the added overhead introduced by using the scalar product protocol is manageable as the optimization step forms a much more significant bottleneck. As such, the runtime duration is comparable to the original Verticox algorithm as well.

While Verticox+ improves the privacy guarantees, a number of practical concerns remain. The scalar product protocol relies on a trusted third party. Additionally there is a theoretical possibility of a malicious party reconstructing an approximation of the data, akin to a gradient leak attack in deep learning settings. These risks can be mitigated by applying multiple layers of security measures, such as offering access to only a small number of trusted researchers. Additionally the relevant legal frameworks also need to be established. The need for such frameworks also serves as a reminder that purely technical privacy preserving solutions are not sufficient to establish the necessary trust needed for any federated learning project.

The need for such frameworks, as well as the time complexity of Verticox+, does limit Verticox+ to certain scenarios. Scalability concerns, as well as the need for trusted third parties and a complexity of creating the necessary legal and infrastructure frameworks, means that Verticox+ is not a great fit for an internet of things scenario with many parties, all of which have an extremely low level of trust. However, in formal settings, where it is easier to vet the parties involved, and where parties have access to the technical infrastructure necessary to deal with the scalability issues, it is a great tool in the federated learning toolbox.

### Summary

In this paper we have proposed an improvement to the Verticox algorithm dubbed Verticox+. Verticox+ brings improved privacy guarantees. Our experiments show that Verticox+ produces the same end-result, without a noticeable change in overhead costs.

## Data Availability

Not applicable.

## Footnotes

1 https://github.com/CARRIER-project/verticox.

## Code availability

the code is available and can be found here: Main algorithm code: https://github.com/CARRIER-project/verticox.

## References

[1] Kairouz P, McMahan HB, Avent B, Bellet A, Bennis M, Bhagoji AN, Bonawitz K, Charles Z, Cormode G, Cummings R, D’Oliveira RGL, Rouayheb SE, Evans D, Gardner J, Garrett Z, Gascón A, Ghazi B, Gibbons PB, Gruteser M, Harchaoui Z, He C, He L, Huo Z, Hutchinson B, Hsu J, Jaggi M, Javidi T, Joshi G, Khodak M, Konečný J, Korolova A, Koushanfar F, Koyejo S, Lepoint T, Liu Y, Mittal P, Mohri M, Nock R, Özgür A, Pagh R, Raykova M, Qi H, Ramage D, Raskar R, Song D, Song W, Stich SU, Sun Z, Suresh AT, Tramèr F, Vepakomma P, Wang J, Xiong L, Xu Z, Yang Q, Yu FX, Yu H, Zhao S (2019) Advances and Open Problems in Federated Learning. arXiv:1912.04977 [cs, stat]. arXiv: 1912.04977. Accessed 2021-03-02

[2] Li L, Fan Y, Tse M, Lin K-Y (2020) A review of applications in federated learning. Comput Ind Eng 149:106854. 10.1016/j.cie.2020.106854. (**Accessed 2021-03-03**)

[3] Dai W, Jiang X, Bonomi L, Li Y, Xiong H, Ohno-Machado L (2020) VERTICOX: vertically distributed Cox proportional hazards model using the alternating direction method of multipliers. IEEE Trans Knowl Data Eng. 10.1109/TKDE.2020.2989301. Accessed 2021-05-26. (**Accessed 2021-05-26**) 10.1109/tkde.2020.2989301 PMC9491599 36158636

[4] Cox DR (1972) Regression models and life-tables. J Roy Stat Soc: Ser B (Methodol) 34(2):187–202. 10.1111/j.2517-6161.1972.tb00899.x. (**Accessed 2024-05-22**)

[5] Boyd S, Parikh N, Chu E, Peleato B, Eckstein J (2011) Distributed optimization and statistical learning via the alternating direction method of multipliers. Found Trends Mach Learn 3(1):1–122. 10.1561/2200000016. (**Accessed 2024-05-21**)

[6] Miao G, Yu L, Yang J, Bennett DA, Zhao J, Wu SS (2024) Learning from vertically distributed data across multiple sites: an efficient privacy-preserving algorithm for Cox proportional hazards model with variable selection. J Biomed Inform 149:104581. 10.1016/j.jbi.2023.104581 38142903 10.1016/j.jbi.2023.104581 PMC10996392

[7] Kamphorst B, Rooijakkers T, Veugen T, Cellamare M, Knoors D (2022) Accurate training of the Cox proportional hazards model on vertically-partitioned data while preserving privacy. BMC Med Inform Decis Mak 22(1):49. 10.1186/s12911-022-01771-3 35209883 10.1186/s12911-022-01771-3 PMC8867891

[8] Yao AC (1982) Protocols for secure computations. In: 23rd Annual Symposium on Foundations of Computer Science (sfcs 1982), pp. 160–164. 10.1109/SFCS.1982.38 . ISSN: 0272-5428

[9] Lu Y, Tian Y, Zhou T, Zhu S, Li J (2021) Multicenter privacy-preserving cox analysis based on homomorphic encryption. IEEE J Biomed Health Inform 25(9):3310–3320 33822728 10.1109/JBHI.2021.3071270

[10] Du W, Zhan Z (2002) Building decision tree classifier on private data. In: Proceedings of the IEEE International Conference on Privacy, Security and Data Mining - Volume 14. CRPIT ’14, pp. 1–8. Australian Computer Society, Inc., AUS

[11] Moncada-Torres A, Martin F, Sieswerda M, Van Soest J, Geleijnse G (2021) VANTAGE6: an open source priVAcy preserviNg federaTed leArninG infrastructurE for Secure Insight eXchange. AMIA Ann Symp Proc 2020:870–877 PMC8075508 33936462

[12] Daalen F, Ippel L, Dekker A, Bermejo I (2023) Privacy Preserving n-Party Scalar Product Protocol. IEEE Trans Parallel Distrib Syst 34(4):1060–1066. 10.1109/TPDS.2023.3238768. (**Conference Name: IEEE Transactions on Parallel and Distributed Systems**)

[13] Shmueli E, Tassa T Mediated secure multi-party protocols for collaborative filtering. 11(2), 1–25 10.1145/3375402. (**Accessed 2022-10-26**)

[14] Goethals B, Laur S, Lipmaa H, Mielikäinen T (2005) On Private Scalar Product Computation for Privacy-Preserving Data Mining. In: Hutchison D, Kanade T, Kittler J, Kleinberg JM, Mattern F, Mitchell JC, Naor M, Nierstrasz O, Pandu Rangan C, Steffen B, Sudan M, Terzopoulos D, Tygar D, Vardi MY, Weikum G, Park C-s, Chee S (eds) Information Security and Cryptology ICISC 2004 vol. 3506, pp. 104–120. Springer, Berlin, Heidelberg. 10.1007/11496618_9 . Series Title: Lecture Notes in Computer Science. Accessed 2021-06-28

[15] Atallah MJ, Du W (2001) Secure Multi-party Computational Geometry. In: Goos, G, Hartmanis J, Leeuwen J, Dehne F, Sack J-R, Tamassia R (eds) Algorithms and Data Structures vol. 2125, pp. 165–179. Springer, Berlin, Heidelberg.10.1007/3-540-44634-6_16 . Series Title: Lecture Notes in Computer Science. Accessed 2021-07-19

[16] Du W, Atallah MJ (2001) Privacy-preserving cooperative statistical analysis. In: Seventeenth Annual Computer Security Applications Conference, pp. 102–110. IEEE Comput. Soc, New Orleans, LA, USA. 10.1109/ACSAC.2001.991526. Accessed 2021-06-16

[17] Vaidya J, Clifton C (2002) Privacy preserving association rule mining in vertically partitioned data. In: Proceedings of the Eighth ACM SIGKDD International Conference on Knowledge Discovery and Data mining. KDD ’02, pp. 639–644. Association for Computing Machinery, New York, NY, USA. 10.1145/775047.775142. Accessed 2021-06-16

[18] Sembay Z (2021) Seer breast cancer data. Zenodo. 10.5281/zenodo.5120960

[19] Harrell FE Jr, Lee KL, Mark DB (1996) Multivariable prognostic models: issues in developing models, evaluating assumptions and adequacy, and measuring and reducing errors. Stat Med 15(4):361–387 10.1002/(SICI)1097-0258(19960229)15:4<361::AID-SIM168>3.0.CO;2-4 8668867

[20] Wang J, Guo S, Xie X, Qi H (2022) Protect privacy from gradient leakage attack in federated learning. In: IEEE INFOCOM 2022—IEEE Conference on Computer Communications, pp. 580–589. IEEE, London, United Kingdom. 10.1109/INFOCOM48880.2022.9796841. Accessed 2024-05-21

[21] Jin X, Chen P-Y, Hsu C-Y, Yu C-M, Chen T (2021) CAFE: catastrophic data leakage in vertical federated learning. In: Advances in Neural Information Processing Systems, vol. 34, pp. 994–1006. Curran Associates, Inc., https://proceedings.neurips.cc/paper_files/paper/2021/hash/08040837089cdf46631a10aca5258e16-Abstract.html. Accessed 2024-05-21

[22] Wei W, Liu L, Loper M, Chow K-H, Gursoy ME, Truex S, Wu Y (2020) A framework for evaluating gradient leakage attacks in federated learning. arXiv. arXiv:2004.10397 [cs, stat]. 10.48550/arXiv.2004.10397 . arxiv:2004.10397 Accessed 2024-05-21

